# α2-macroglobulin-rich serum as a master inhibitor of inflammatory factors attenuates cartilage degeneration in a mini pig model of osteoarthritis induced by “idealized” anterior cruciate ligament reconstruction

**DOI:** 10.3389/fphar.2022.849102

**Published:** 2022-09-05

**Authors:** Ruipeng Zhao, Xiaochun Wei, Chengming Zhang, Hongru Wu, Chuan Xiang, Haoqian Li, Wangping Duan, Zhiqing Duan, Chunjiang Li, Yu Zhao, Lingan Huang

**Affiliations:** ^1^ Department of Orthopaedics, The Second Hospital of Shanxi Medical University. Shanxi Key Laboratory of Bone and Soft Tissue Injury Repair, Taiyuan, China; ^2^ Shanxi Institute of Sports Science, Taiyuan, China; ^3^ Department of Biochemistry and Molecular Biology, School of Basic Medical Sciences, Shanxi Medical University, Taiyuan, China; ^4^ Department of Pain Medicine, Sanya Central Hospital of Hainan Medical College, Sanya, China

**Keywords:** osteoarthritis, α2M-rich serum, anterior cruciate ligament, articular cartilage, inflammation, mini pig

## Abstract

Post-traumatic osteoarthritis is a special type of osteoarthritis and a common disease, with few effective treatments available. α2-Macroglobulin (α2M) is important to chondral protection in post-traumatic osteoarthritis. However, its injection into xenogeneic joint cavities involves safety hazards, limiting clinical applications. Exploring serum α2M-enriching strategies and the therapeutic effect and mechanism of α2M-rich serum (α2MRS) autologous joint injection to treat post-traumatic osteoarthritis has significant value. In the present study, a unique filtration process was used to obtain α2MRS from human and mini pig serum. We evaluated the potential of α2MRS in protecting against post-surgery cartilage degeneration. We identify the potential of α2MRS in reducing the expression of inflammatory cytokines and factors that hasten cartilage degeneration in post-operative conditions leading to post-traumatic osteoarthritis. The potential of α2MRS was analyzed in interleukin-1β induced human chondrocytes and mini pig models. In the chondrocyte model, α2MRS significantly promoted human chondrocyte proliferation and reduced apoptosis and chondrocyte catabolic cytokine gene transcription and secretion. The anterior cruciate ligament autograft reconstruction model of mini pigs was randomized into groups, operated on, and injected with α2MRS or saline. The results showed that α2MRS injection significantly suppressed the levels of inflammatory factors, improved gait, and showed significantly lower cartilage degeneration than the groups that did not receive α2MRS injections. This study highlights the chondroprotective effects of α2MRS, elucidated its potential applications against cartilage degeneration, and could provide a basis for the clinical translation of α2MRS.

## Introduction

Anterior cruciate ligament (ACL) rupture, one of the most common joint injuries in young people, is conventionally treated using surgical ACL reconstruction (ACL-R). However, even with the best surgical techniques available, these patients remain at a high risk for post-traumatic osteoarthritis (PTOA) (Barenius et al., 2014; Björnsson et al., 2016; Wang et al., 2020). Recently, researchers developed an “idealized” ACL autograft reconstruction (IACL-R) model (Bryan et al., 2011; Han et al., 2018). Notably, the authors found that cartilage degeneration still occurred despite this reconstruction and concluded that there was a significant correlation between the expression of inflammatory factors and cartilage injury. Moreover, other studies have indicated that catabolic proteases and cytokines reach their peak levels within 48 h after joint injury, initiating cell death and cartilage matrix degeneration (Lieberthal et al., 2015; Heard et al., 2016; Maerz et al., 2018). Thus, early intervention to reduce the expression of these catabolic proteases and cytokines is critical to prevent or delay cartilage degeneration.

α2-macroglobulin (α2M), a tetrameric macromolecular glycoprotein, is mainly synthesized and secreted into the body fluids by liver epithelial parenchymal cells (Rehman et al., 2013). To date, α2M has been shown to play an important role in the diagnosis of diseases, prediction of liver fibrosis staging (Ho et al., 2010), non-invasive diagnosis of type II diabetes (Chung et al., 2016), and the treatment of various diseases, including alleviating pain in subacromial bursitis, lateral epicondylitis, Achilles tendonitis, spinal intervertebral discogenic (Montesano and Cuellar, 2015; Cuellar et al., 2016), and jaw osteoradionecrosis (Li S. et al., 2019). Moreover, some studies have demonstrated that supplemental intra-articular α2M provides chondral protection in PTOA (Wang et al., 2014; Zhang et al., 2017; Li et al., 2019). However, α2M is expensive. More importantly, the long-term injection of α2M, a blood protein component, into the xenogeneic joint cavity involves safety hazards, such as immune rejection, which limits its clinical application. This study used ultrafiltration centrifugation to explore suitable centrifugation conditions, in an attempt to enrich α2M in serum, and finally prepare α2M-rich serum (α2MRS). The ultimate purpose of the α2MRS preparation is for clinical application and disease prevention and treatment. Therefore, its biological safety, efficacy, and specific mechanism of action need to be accurately evaluated and verified. We hypothesized that α2MRS could significantly reduce the expression of inflammatory factors in synovial fluid, promote early recovery of the gait, and effectively attenuate cartilage degeneration. This study will greatly promote the translation of α2MRS for clinical applications.

## Materials and methods

### Patient samples and experimental animals

All procedures in this study, including *in vitro* and *in vivo* experiments, were approved by the Ethics Committee of the Second Hospital of Shanxi Medical University (NO. SYDL2019001). Human chondrocytes used in this study were derived from the articular cartilage library of Shanxi Key Laboratory of Bone and Soft Tissue Injury Repair. All patients provided informed consent, and all procedures were approved by the Ethics Committee of the Second Hospital of Shanxi Medical University (NO. 2019YX260). Based on the purpose of this study, under the guidance of statistical experts, the number of samples numbersin this study was six. For the *in vitro* experiments, a sample size of six was used. For the *in vivo* experiments, the number of animals in each group was determined as six. Patient baseline characteristics were as follows: age, 64 ± 3.38 (years); sex, female (*n* = 4)/male (*n* = 2); height, 163 ± 3.53 (cm); weight: 65 ± 3.85 (kg). Mini pigs were purchased from the Beijing Shichuang Century Mini pig Breeding Base (Certificate number: SCXX (jing)2013-0008). All animals were housed at the China Institute for Radiation Protection (Certificate number: SYXK (Jin)2016-0002).

### Reagents

The following antibodies were used in this study: collagen-2 (Col-2, ab34712, Abcam), matrix metalloproteinase -3 (MMP-3, bs-0413R, Bioss), MMP-9 (bs-4593R, Bioss), MMP-13 (K009743P, Solarbio), Col-10 (bs-0554R, Bioss), and Runt-related transcription factor 2 (Runx-2, ab76956, Abcam).

### α2M concentrate from human serum

Whole blood (13 ml) was collected in a coagulation tube (BD Vacutainer SSTTM II,United States ) and centrifuged at 2,000 × g for 20 min to obtain 6 ml of serum, which was then added to the upper filter of the ultrafiltration tube (Cytonics Corporation, West Palm Beach, Florida, United States ). The upper concentrate was obtained under different conditions of centrifugal force (3,000, 4,000, and 5,000 × *g*) and time (20, 30, and 40 min). Finally, the best concentration conditions were determined based on the concentration of α2M in the upper concentrate.

### Human chondrocyte isolation and primary culture

Human chondrocytes were isolated as previously described (Guo et al., 2019) and plated in 6-well culture plates at a density of 1 × 10^6^ cells/plate. Chondrocytes culture medium was Dulbecco’s Modified Eagle Medium/Nutrient Mixture F-12 (DMEM/F-12) containing 10% fetal bovine serum (Hyclone) At 90% confluence, the cells were cultured overnight under serum-free conditions and then treated with 10 ng/ml recombinant human interleukin (IL)-1β for 2 h before treatment with α2MRS. It was ensured that the concentration of α2M was 0.25 mg/ml in the culture medium. The culture medium and chondrocytes were collected and analyzed.

### Elisa assays

α2M concentrations in the upper concentrate under different centrifugal conditions were determined using ELISA (EK1118, Boster Bio, China). The human chondrocyte culture medium was collected 24 h after α2MRS treatment and analyzed for the presence of MMP-13, tumor necrosis factor-α (TNF-α), and IL-6 using ELISA.

### Human chondrocyte proliferation and apoptosis assays

Human chondrocyte proliferation was detected at 0, 24, 36, and 48 h using the Cell Counting Kit-8 (CCK-8) cell viability assay kit (Boster Biological Technology, China). Human chondrocytes were collected 24 h after α2MRS treatment and apoptosis was detected using a Terminal transferase dUTP end Labeling (TUNEL) assay kit (Key GEN Bio TECH, China). The percentage of positive cells was determined. The detailed procedure was in accordance with the manufacturer’s protocol.

### RNA isolation and real-time PCR assays

mRNA levels of *col-2, aggrecan, MMP-3*, and *MMP-13* in human chondrocyte samples and those plus *collagen-10 a1 (col-10 a1)* and *Runx-2* in minipig cartilage weight-bearing sites of the medial tibial plateau were measured by real-time PCR. Primer pairs are listed in Supplementary Table S1. Levels of gene expression were normalized to 18S rRNA expression. The data were analyzed using the comparison Ct (2^−ΔΔCt^) method and expressed as the fold-change relative to the respective control. The detailed PCR procedure was described previously (Gu et al., 2019).

### Idealized ACL autograft reconstruction surgery

**FIGURE 1:**
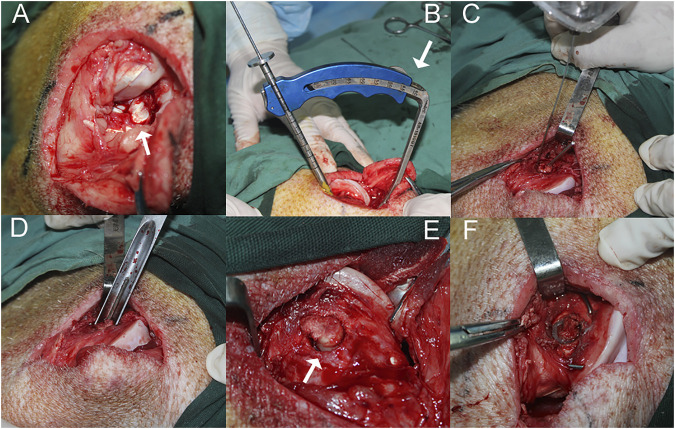
The surgical procedure performed on the right hind limb of a minipig in the IACL-R and IACL-R+α2MRS groups. **(A)** The stifle joint was open and the patella was dislocated to expose the ACL (arrow). **(B)** The ACL reconstruction guide was positioned at a 45° angle (arrow) to the longitudinal axis of the femur. **(C)** Before the hollow drill was about to penetrate the femoral tunnel, a Kirschner wire (diameter 1 mm) was drilled along outer edge of the tunnel to prevent the cartilage from splitting. **(D)** The tunnel was gently penetrated by the same diameter thin-walled ring osteotomy to completely severv tendon-bone segment **(E)** The tendon-bone segment was completely freed (arrow). **(F)** The tendon-bone segment was fixed *in situ* with two crossed Kirschner wires.

Eighteen mature female mini pigs (age, 18 ± 1.55 months; weight, 43.3 ± 3.67 kg) were randomized into three groups based on animal ear numbers: sham (*n* = 6), IACL-R (*n* = 6), and IACL-R+α2MRS (*n* = 6). All surgeries were performed under anesthesia via an intramuscular injection of 25 mg/ml tiletamine and 25 mg/ml zolazepam (Zoletil 50, 1 ml/15 kg; Virbac Group, Carros, France). Unilateral surgery was performed on the right hind limbs of all mini pigs. Mini pigs in the IACL-R and IACL-R+α2MRS groups were subjected to surgery based on methods described previously (Figure 1; Bryan et al., 2011; Han et al., 2018). The minipigs in the sham group underwent arthrotomy, temporary patellar dislocation, and coring of one-third of the length of the lateral femoral condyle. Specific information on animal care can be found in the Supplementary Text S1.

### Mini pig α2MRS reserve

With the help of a veterinarian, 120 ml of whole blood was collected from the anterior vena cava of each mini pig into coagulation tubes before IACL-R. According to the best concentration conditions (centrifugal force: 5,000 × *g*; time: 30 min), 12–15 ml of α2MRS was obtained per mini pig (marked according to the mini pig ear number), and these samples were frozen at −80°C.

### Synovial fluid collection

Synovial fluid from the right hind limbs of all animals was collected preoperatively (day 0) and postoperatively on days 3, 6, 14, 29, and 90. The detailed procedure was described previously (Wei et al., 2010).

### Intra-articular injections

Under general anesthesia, intra-articular injections were administered 2, 6, 14, and 29 days post-surgery using a sterile syringe. Under aseptic conditions, 2.5 ml autologous α2MRS was injected into the right hind limbs of mini pigs in the IACL-R+α2MRS group on the indicated days. Animals in the sham and IACL-R groups were administered an equivalent volume of saline.

### Luminex assay

The Millipore Porcine Cytokine Magnetic Bead Panel (EMD Millipore, No. PCYTMAG-23K) was used to measure the levels of IL-1α, IL-1β, IL-2, IL-6, IL-8, IL-18, TNF-α, and granulocyte-macrophage colony-stimulating factor (GM-CSF). Luminex assays were performed as previously described (Han et al., 2018).

### Gait assessment

Six gait indicators related to biomechanics—maximum force, contact area, peak force, impulse, stance time, and swing time—were determined using the Tekscan Walkway system (Tekscan Inc., United States ) (Rashid et al., 2013; Shah et al., 2020). To rule out individual differences in learning skills and walking states, each animal was subjected to over ten successful training sessions per day for ten consecutive days before the surgery, and all indicators were expressed as the ratio of the average values for the left hind limb divided by the average values for the right hind limb (Ruan et al., 2013). Gait data were collected preoperatively (day 0) and postoperatively on days 7, 15, 30, 45, 60, 75, and 90. All results obtained were the average of five successful repeated walkway trials performed at each time point for each animal.

### Imaging assessment

Three months after the surgery, the mini pigs were euthanized with a pentobarbital overdose, and their right hind limbs were severed from the hip joint. Each right hind limb semi flexed was immediately subjected to X-ray examination, computed tomography (CT), three-dimensional CT reconstruction (3D CT), and magnetic resonance imaging (MRI). The specific imaging parameters are listed in Supplementary Text S2.

We determined the Kellgren-Lawrence grade of the right hind limb of each animal by examining the X-ray image (Kellgren and Lawrence, 1957; Misir et al., 2020). The CT values and thickness of the subchondral bone plate were determined (Hu et al., 2020). To avoid interference by metal artifacts, we obtained CT scans of only the middle sagittal plane from the medial femoral condyle and medial tibial plateau of the right hind limb. We also determined the whole-organ MRI score (WORMS) of the medial femoral condyle and medial tibial plateau of the right hind limb (Pozzi et al., 2015; Xue et al., 2021).

### Macroscopic cartilage and osteophyte assessment

Macroscopic damage to the articular cartilage surfaces and osteophyte formation on the medial femoral condyle, medial tibial plateau, lateral femoral condyle, lateral tibial plateau, and trochlea were scored according to the Osteoarthritis Research Society International (OARSI) recommendations for sheep and goats (Little et al., 2010)

### Histological assessment

Cartilage samples were obtained by drilling (φ8 mm; MOC Medizinische Geräte Gmbh, Fedderingen, Germany) the weight bearing site of the medial femoral condyle. The cartilage tissue sections (6 µm) were stained with safranin O and fast green as previously described and scored according to the recommendations of OARSI (Little et al., 2010). Furthermore, we collected synovium samples from inside the joint capsule. Synovium sections (4 µm) were stained with hematoxylin and eosin (H&E) as previously described and scored according to the OARSI recommendations (Little et al., 2010). Vertical meniscus slices from the middle region of the medial meniscus were processed and stained using H&E and scored according to the protocol detailed by Pauli et al. (2011).

### Immunohistochemical assessment

Immunohistochemical analysis was conducted as reported previously (Li et al., 2020). Briefly, to detect the distribution of the target protein, 6-μm thick cartilage tissue sections from the medial femoral condyle were collected on positively-charged glass slides. Endogenous peroxidase was blocked by treating the sections with 3% hydrogen peroxide in methanol. The sections were incubated with specific antibodies against Col-2 (1:500), MMP-3 (1:200), MMP-9 (1:100), MMP-13 (1:100), Col-10 (1:50), and Runx-2 (1:50) at 4 °C overnight. Thereafter, the sections were treated sequentially with a biotinylated secondary antibody and streptavidin–peroxidase conjugate and then developed with 3,3′-diaminobenzidine chromogen. Quantitative immunohistochemical analysis was performed using an imaging analyzer.

### Statistical analysis

The SPSS statistical software (version 13.0) was used to analyze the collected data. Differences in gaits and inflammatory factor levels between the preoperative (day 0) and postoperative time points in the same group were analyzed using multiple comparisons of repeated measurement data. Differences in human chondrocytes, minipig gait, inflammatory factor levels, CT values, thicknesses of the subchondral bone plate T2 values, and quantitative immunohistochemical analysis at the same time point among the different groups were estimated using one-way analysis of variance. Differences in macroscopic cartilage and osteophyte scores; microscopic cartilage, synovium, and meniscus scores; and WORMS were estimated using nonparametric tests (Wilcoxon rank-sum test). Differences in Kellgren-Lawrence grades were estimated using Fisher probabilities. Statistical significance was set at *p* < 0.05.

## Results

All animals recovered from anesthesia and were fully awake within 3-5 h after the surgery. There were no instances of infection or immune rejections.

### Concentration analysis of α2M in different concentrates from human and mini pig

**FIGURE 2:**
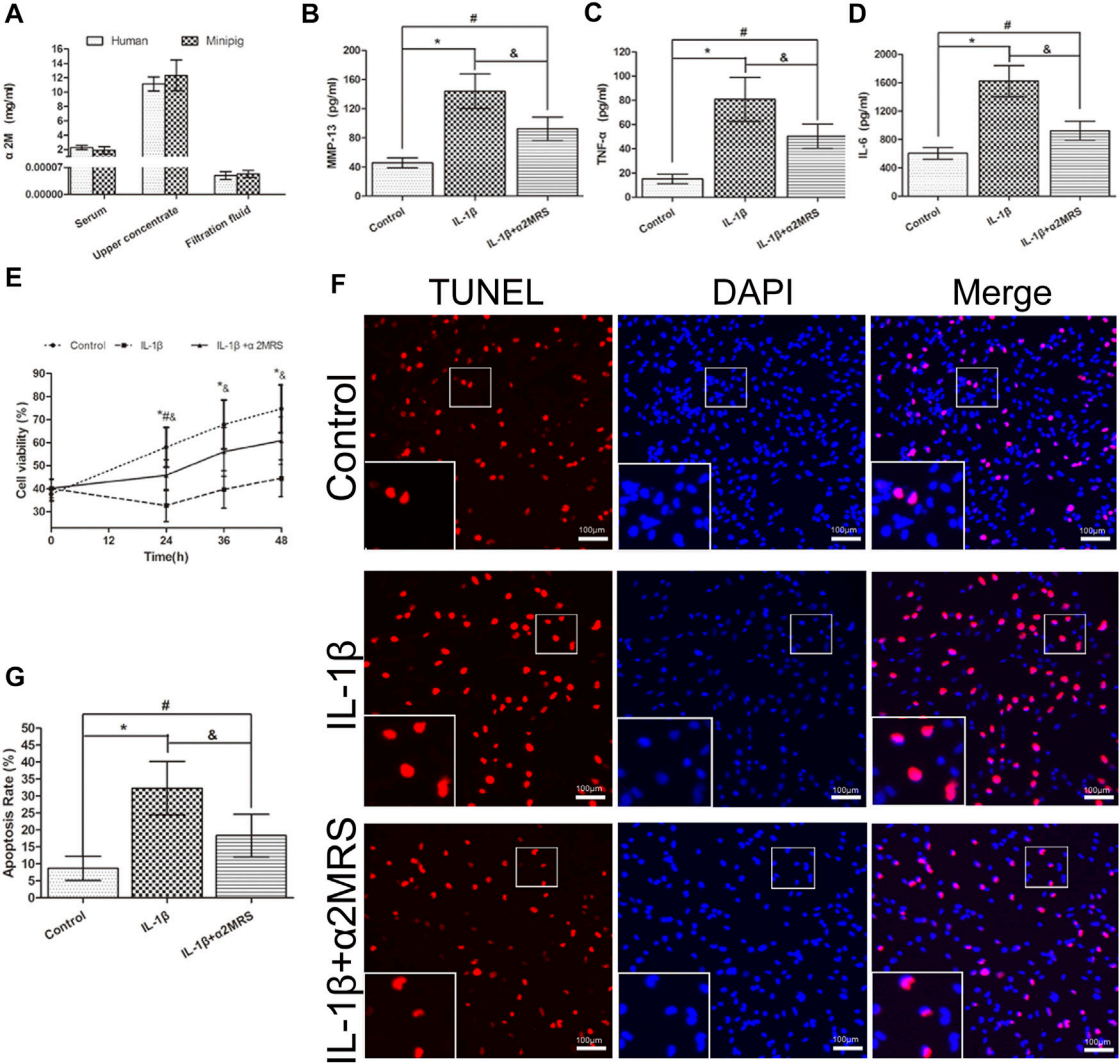
**(A)**. Human and mini pig α2M concentrations after centrifugation at 5,000 *g* for 30 min. Human: serum: 2.28 ± 0.26 mg/ml; upper concentrate: 11.13 ± 0.90 mg/ml; filtration fluid: 0.000049 ± 0.00001 mg/ml; mini pigs: serum: 1.90 ± 0.46 mg/ml; upper concentrate: 12.32 ± 1.97 mg/ml; filtration fluid: 0.000053 ± 0.000008 mg/ml. **(B–D)**. α2MRS significantly inhibited the induction of MMP-13,TNF-α and IL-6 in IL-1β induced human primary osteoarthritic chondrocytes. **(E)** The CCK-8 assay results showed that the viability of chondrocytes was higher in IL-1β+α2MRS group relative to that in the IL-1β group, and the viability gradually increased with a longer treatment time. **(F)** TUNEL assay results showed that apoptosis (red) was reduced in the IL-1β+α2MRS group seem to that in the IL-1β group. The bottom panels are higher-magnification views of the boxed areas in the top panels **(G)** The percentage of TUNEL-positive cells was quantified, and the apoptosis rate of chondrocytes was significantly reduced in the IL-1β+α2MRS group relative to that in the IL-1β group. * = *p* < 0.05, control group versus IL-1β group; # = *p* < 0.05, control group versus IL-1β+α2MRS group; and = *p* < 0.05, IL-1β group versus IL-1β+α2MRS group. The bars show the mean ± SD (*n* = 6).

As the centrifugal force increased and centrifugation time was prolonged, human α2M concentrations in the upper concentrate increased correspondingly (Table 1). Considering different factors, such as protein biological activity and concentrate volume, the concentration effect of α2M under the conditions of centrifugation at 5,000 *g* for 30 min was ideal. For human α2MRS, the concentration of α2M was 11.13 mg/ml, which was 4.88-fold higher than that in normal human serum. In mini pigs, the concentration of α2M was 12.32 mg/ml, which was 6.48-fold higher than that in normal pig serum (Figure 2A).

**TABLE 1:** Human α2M concentrations in the upper concentrate under different centrifugal conditions (mg/ml) (Mean ± SD, *n* = 6).

	3,000 × *g*	4,000 × *g*	5,000 × *g*
20min	4.82 ± 0.75	6.98 ± 1.15	8.23 ± 1.12
30min	6.46 ± 0.89	9.22 ± 1.08	11.13 ± 0.90
40min	7.39 ± 0.98	9.92 ± 1.17	11.93 ± 1.53

### Human chondrocyte culture medium analysis

ELISA results demonstrated that exogenous α2MRS significantly inhibited the induction of MMP-13 (*p* = 0.001), TNF-α (*p* = 0.005), and IL-6 (*p* < 0.001) activity in IL-1β-induced human primary osteoarthritis chondrocytes (Figures 2B–D).

### Human chondrocyte proliferation and apoptosis analysis

α2MRS promoted the proliferation and reduced the apoptosis of human chondrocytes *in vitro.* The results of the CCK-8 assay showed that the viability of chondrocytes was higher in the IL-1β+α2MRS group than in the IL-1β group (*p* < 0.001), and the viability gradually increased with treatment time (Figure 2E). The results of the TUNEL assay showed that apoptosis was significantly reduced in the IL-1β+α2MRS group (18.33% ± 5.71%) relative to that in the IL-1β group (32.33% ± 7.23%; *p* < 0.001; Figures 2F,G).

### Real-time PCR analysis

**FIGURE 3:**
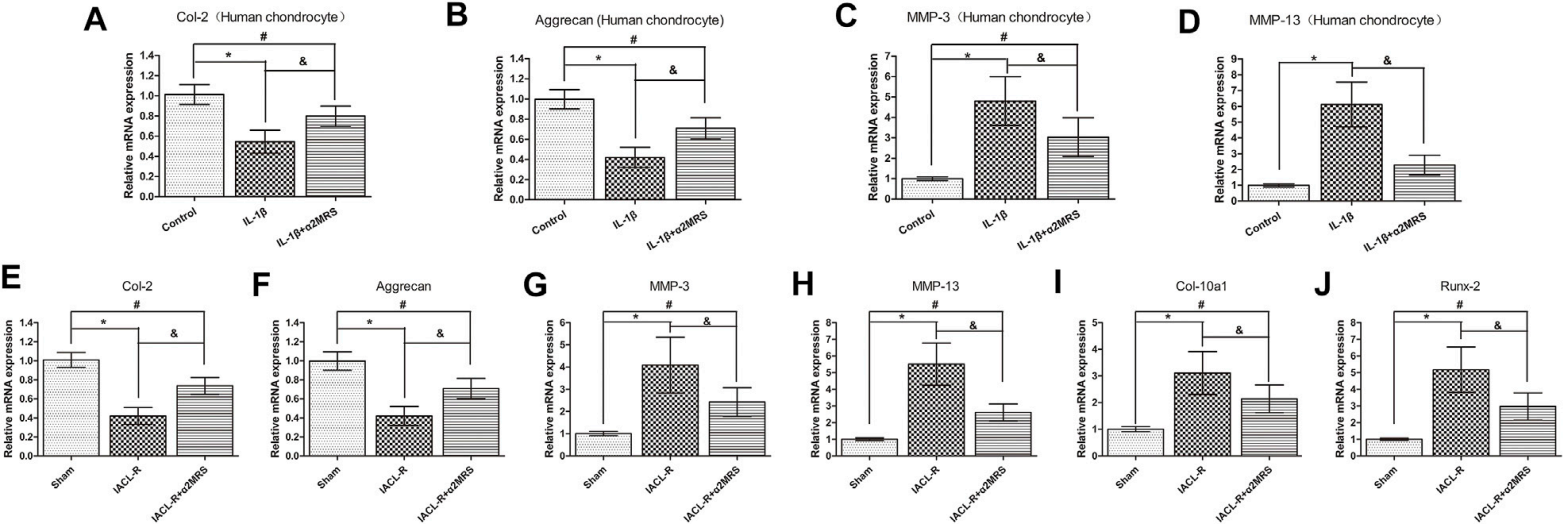
Real-time PCR data indicated that supplementation with α2MRS reduced cartilage matrix catabolism and enhanced anabolism *in-vitro* **(A–D)** and *in-vivo* **(E–J)**. mRNA levels of *MMP-3* (*p* < 0.001), *MMP-13* (*p* < 0.001), *Col-10* a1 (*p* = 0.001), and *Runx-2* (*p* < 0.001)were lower in IL-1β+α2MRS and IACL-R+α2MRS groups than in IL-1β and IACL-R groups respectively. In contrast, mRNA levels of *Col-2* (*p* < 0.001) and *aggrecan* (*p* < 0.001) followed the opposite pattern. Both were increased in IL-1β+α2MRS and IACL-R+α2MRS groups as compared to levels in IL-1β and IACL-R groups. * = *p* < 0.05, control group versus IL-1β group or sham group versus IACL-R group; # = *p* < 0.05, control group versus IL-1β+α2MRS group or sham group versus IACL-R+α2MRS group; and = *p* < 0.05, IL-1β group versus IL-1β+α2MRS group or IACL-R group versus IACL-R+α2MRS group. The bars show the mean ± SD (*n* = 6).

Real-time PCR data indicated that supplementation with α2MRS reduced cartilage matrix catabolism and enhanced anabolic metabolism *in vitro* (Figures 3A–D) and *in vivo* (Figures 3E–J). mRNA levels of *MMP-3* (*p* < 0.001), *MMP-13* (*p* < 0.001), *Col-10 a1* (*p* = 0.001), and *Runx-2* (*p* < 0.001) were lower in the IL-1β+α2MRS and IACL-R+α2MRS groups than in the IL-1β and IACL-R groups. In contrast, mRNA levels of *Col-2* (*p* < 0.001) and *aggrecan* (*p* < 0.001) showed the opposite pattern. Both were increased in the IL-1β+α2MRS and IACL-R+α2MRS groups as compared to the levels in the IL-1β and IACL-R groups, respectively.

### Inflammatory factor analysis

**FIGURE 4:**
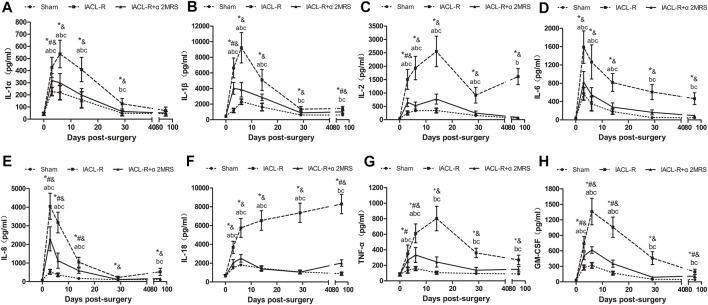
Line charts of inflammatory factors in synovial fluid. **(A)**: IL-1α; **(B)** IL-1β; **(C)** IL-2; **(D)** IL-6; **(E)** IL-8; **(F)** IL-18; **(G)** TNF-α; **(H)** GM-CSF. * = *p* < 0.05, sham group versus IACL-R group at the same time point; # = *p* < 0.05, sham group versus IACL-R+α2MRS group at the same time point; and = *p* < 0.05, IACL-R group versus IACL-R+α2MRS group at the same time point. a = *p* < 0.05, preoperative (day 0) versus postoperative (days 3, 6, 14, 29, and 90) in the sham group; b = *p* < 0.05, preoperative (day 0) versus postoperative (days 3, 6, 14, 29, and 90) in the IACL-R group; c = *p* < 0.05, preoperative (day 0) versus postoperative (days 3, 6, 14, 29, and 90) in the IACL-R+α2MRS group. The bars show the mean ± SD (n = 6)

Postoperatively, changes in the levels of inflammatory factors in different groups, except for IL-18 in the IACL-R group, showed similar trends; the levels markedly increased in the early stage and then decreased significantly. The IL-2 concentration in the IACL-R group subsequently showed an increasing trend from day 30 to day 90. The concentrations of inflammatory factors, including IL-1β, IL-6, IL-18, TNF-α, and GM-CSF, in the IACL-R and IACL-R+α2MRS groups after surgery were significantly higher than those before surgery (*p* < 0.001). The concentration of all tested inflammatory factors other than IL-1α after surgery was significantly lower in the IACL-R+α2MRS group than in the IACL-R group, and a significant difference in peak concentrations was observed in all factors (*p* < 0.001). Moreover, the peak concentrations of all detected inflammatory factors, other than IL-18 in the IACL-R group, appeared within 3–14 days after surgery (Figure 4).

### Gait assessment

**FIGURE 5:**
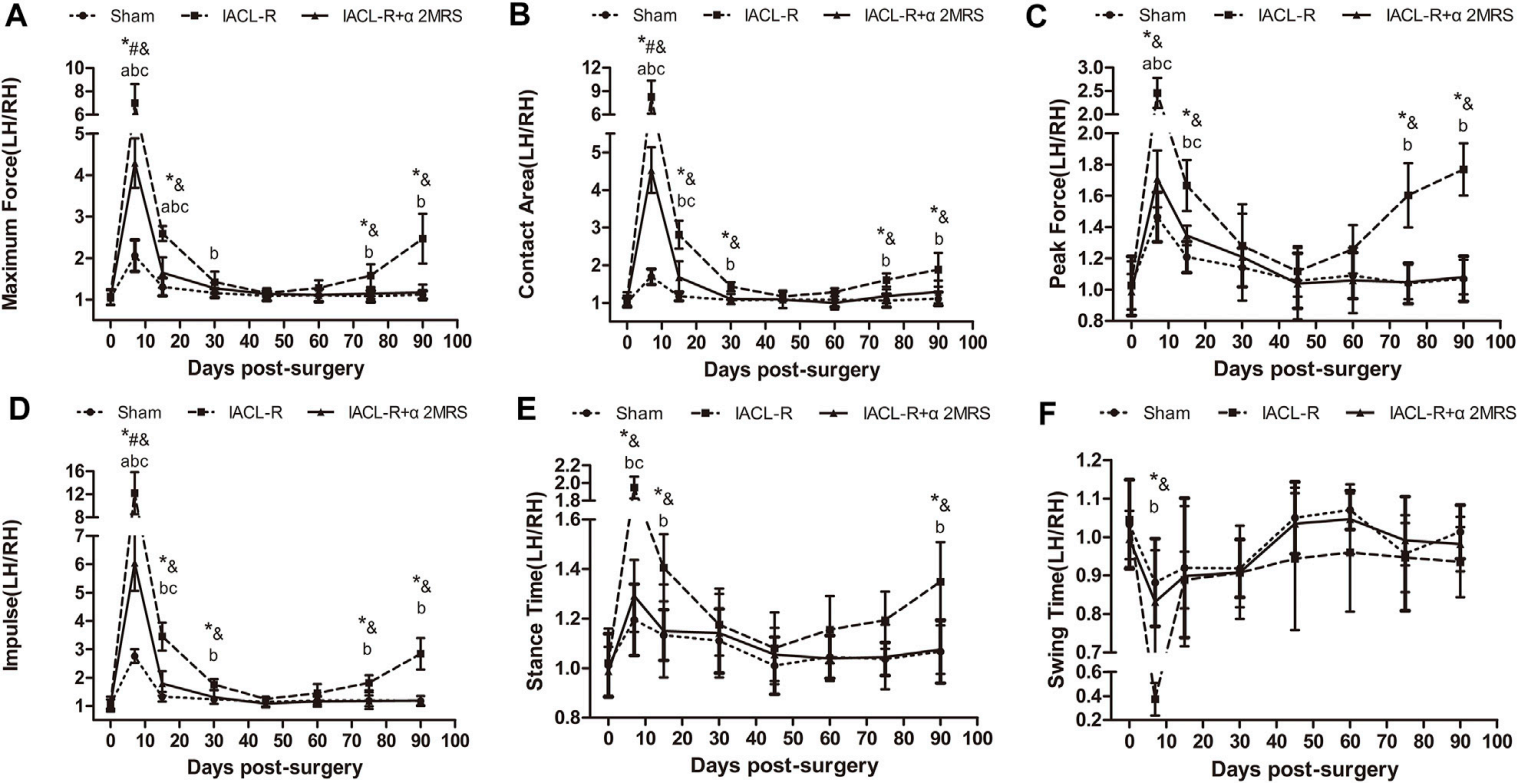
Line charts of gait analysis. **(A)**Maximum force; **(B)**Contact area; **(C)**Peak force; **(D)**Impulse; **(E)**Stance time; **(F)** Swing time. * = *p* < 0.05, sham group versus IACL-R group at the same time point; # = *p* < 0.05, sham group versus IACL-R+α2MRS group at the same time point; and = *p* < 0.05, IACL-R group versus IACL-R+α2MRS group at the same time point. a = *p* < 0.05, preoperative (day 0) versus postoperative (days 7, 15, 30, 45, 60, 75, and 90) in the sham group; b = *p* < 0.05, preoperative (day 0) versus postoperative (days 7, 15, 30, 45, 60, 75, and 90) in the IACL-R group; c = *p* < 0.05, preoperative (day 0) versus postoperative (days 7, 15, 30, 45, 60, 75, and 90) in the IACL-R+α2MRS group. The bars show the mean ± SD (n = 6)

Across all groups, the ratios of all gait parameters of the left hind limb to those of the right hind limb initially showed an increasing trend, followed by a decreasing trend, except in the IACL-R group, which showed another increasing trend toward the end. In the IACL-R group, no gait parameters, from day 45 until day 75, differed significantly from those on day 0 (*p* > 0.05). In the IACL-R+α2MRS group, no gait parameters, from day 30 until euthanasia, differed significantly from those on day 0 (*p* > 0.05) (Figure 5).

The ratios of the left hind limb to the right hind limb of all gait parameters were similar and close to one for symmetry and did not differ significantly in all groups before surgery (*p* > 0.05). Meanwhile, the postoperative ratios of the left hind limb to the right hind limb of the gait parameters were significantly greater than one in all groups, and the values in the IACL-R group were significantly greater than those in the other two groups other than swing time, especially on days 7 and 15 (*p* < 0.001). The ratios of the left hind limb to the right hind limb of the gait parameters were close to one on days 45 and 60, indicating that there were no significant differences among the groups (*p* > 0.05). On day 75, this ratio increased only in the IACL-R group whereas it remained constant in the sham and IACL-R+α2MRS groups. On day 90, all gait parameters other than swing time (*p* = 0.345) significantly differed between the IACL-R group and the other two groups (*p* < 0.001; Figure 5).

### Imaging assessment

**FIGURE 6:**
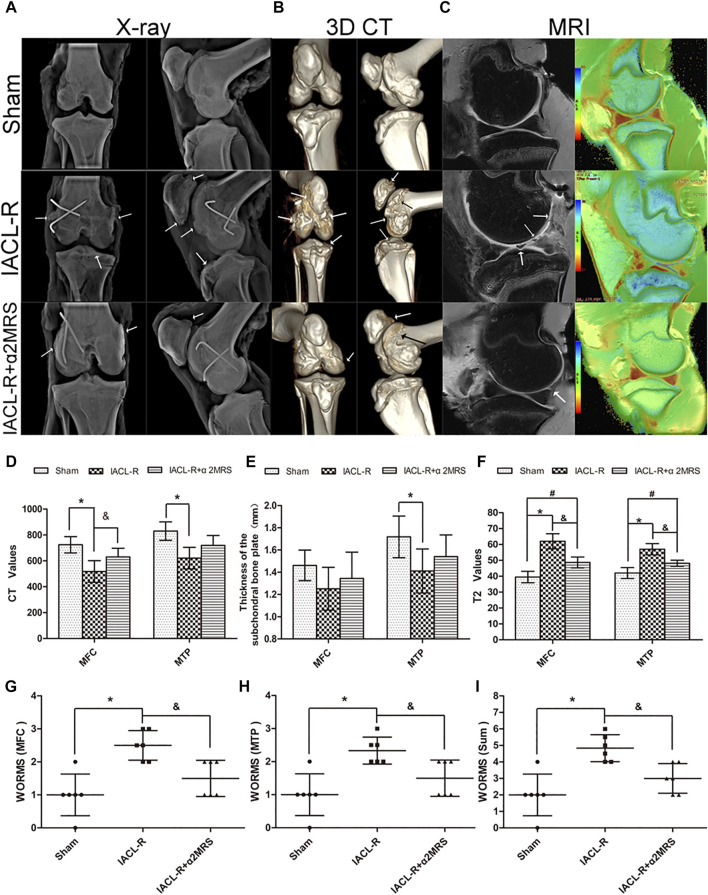
Imaging assessments **(A)**: X-ray examination;(**B)**: three-dimensional CT reconstruction;(**C)**: MRI examination (left: Osag fs PD; right: Osag T2MAP). **(D)**: The CT values of the subchondral bone plate. sham group (medial femoral condyle, 724.2 ± 62.95; medial tibial plateau, 830.3 ± 71.26); IACL-R group (medial femoral condyle, 517.7 ± 83.89; medial tibial plateau, 621.2 ± 83.57); IACL-R+α2MRS group (medial femoral condyle, 630 ± 66.9; medial tibial plateau, 719.2 ± 76.73). **(E)**: The thickness of the subchondral bone plate. sham group (medial femoral condyle, 1.46 ± 0.14; medial tibial plateau, 1.72 ± 0.19); IACL-R group (medial femoral condyle, 1.25 ± 0.19; medial tibial plateau, 1.41 ± 0.20); IACL-R+α2MRS group (medial femoral condyle, 1.35 ± 0.24; medial tibial plateau, 1.54 ± 0.19). **(F)**: Quantification of the T2 values obtained using MRI sag T2MAP.Sham group (medial femoral condyle, 39.5 ± 3.62; medial tibial plateau, 42 ± 3.41); IACL-R group (medial femoral condyle, 62 ± 4.78; medial tibial plateau, 57 ± 3.58); IACL-R+α2MRS group (medial femoral condyle, 48.67 ± 3.45; medial tibial plateau, 48.17 ± 1.94). **(G–I)**: Quantification of the MRI OSag fs PD results using the WORMS guidelines. The white arrows indicate irregularities, osteophytes, and cartilage defects. * = *p* < 0.05, sham group versus IACL-R group; # = *p* < 0.05, sham group versus IACL-R+α2MRS group; and = *p* < 0.05, IACL-R group versus IACL-R+α2MRS group. The bars show the mean ± SD (*n* = 6).

The X-ray examinations showed that joint degeneration in the IACL-R group was relatively noticeable. The joints had a blurred border and mild osteophyte formation compared with those in the IACL-R+α2MRS group (Figure 6A). The Kellgren-Lawrence grades did not significantly differ among the three groups (*p* > 0.05; Table 2). Three-dimensional CT reconstruction showed that all joint surfaces were relatively smooth and flat in the IACL-R+α2MRS group compared to the IACL-R group. Osteophyte was seen on both sides of the patellofemoral joint in the IACL-R group (Figure 6B). Both the medial femoral condyle (*p* < 0.001) and medial tibial plateau (*p* < 0.001) showed significant differences in CT values in the subchondral bone plate between the sham and IACL-R groups. In addition, significant differences in CT values were found between the IACL-R+α2MRS and IACL-R groups in the medial femoral condyle (*p* = 0.028; Figure 6D). Significant differences in the thickness of the subchondral bone plate were found only between the sham and IACL-R groups in the medial tibial plateau (*p* = 0.020; Figure 6E). MRI OSag-fs PD showed that cartilage continuity was better without obvious local defects in the IACL-R+α2MRS group than in the IACL-R group (Figure 6C, left). WORMS of the medial femoral condyle (*p* = 0.006), medial tibial plateau (*p* = 0.014), and sum (*p* = 0.004) were significantly lower in the IACL-R+α2MRS group than in the IACL-R group (Figures 6G–I; Supplementary Table S2). Osag T2MAP showed that regular orange-red layers were more obvious in the IACL-R+α2MRS group than in the IACL-R group (Figure 6C, right). The T2 values of the medial femoral condyle (*p* < 0.001) and medial tibial plateau (*p* < 0.001) were significantly lower in the IACL-R+α2MRS group than in the IACL-R group (Figure 6F).

**TABLE 2:** Kellgren-Lawrence grades on X-ray examination at 3 months (*n* = 6).

Group	Grade 0	Grade 1	Grade 2	Grade 3	Grade 4	P
sham	3	2	1	0	0	
IACL-R	0	1	3	2	0	>0.05
IACL-R+α2M	2	3	1	0	0	

### Macroscopic cartilage and osteophyte assessment

**FIGURE 7:**
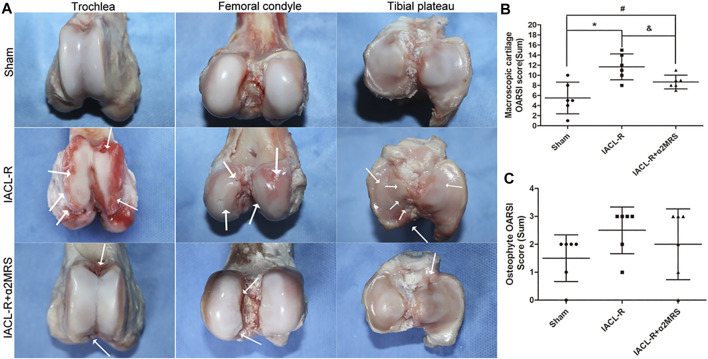
**(A)**: Images of the trochlea, femoral condyle, and tibial plateau for the macroscopic cartilage and osteophyte assessment according to OARSI guidelines. The white arrows indicate cartilage damage, irregularities, and osteophytes. **(B)**: Macroscopic cartilage score were lower in the IACL-R+α2MRS group than in the IACL-R group (*p* = 0.031). **(C)**: Macroscopic osteophyte scores did not differ among the different groups (*p* = 0.438). * = *p* < 0.05, sham group versus IACL-R group; # = *p* < 0.05, sham group versus IACL-R+α2MRS group; and = *p* < 0.05, IACL-R group versus IACL-R+α2MRS group. The bars show the mean ± SD (*n* = 6).

Compared to that in the IACL-R group, cartilage degeneration was relatively low and no obvious cartilage defects or large erosions were found in the IACL-R+α2MRS group (Figure 7A). OARSI scores of macroscopic cartilage were significantly lower in the IACL-R+α2MRS group than in the IACL-R group (*p* = 0.031; Figure 7B; Supplementary Figures S1A–E; Supplementary Table S2). Mild irregular protrusions were found only on sides of the trochlea in the IACL-R group (Figure 7A). No differences were found in the OARSI sum scores of the osteophyte among the three groups (*p* = 0.438; Figure 7C; Supplementary Figures S1F–J; Supplementary Table S2).

### Histological assessment

**FIGURE 8:**
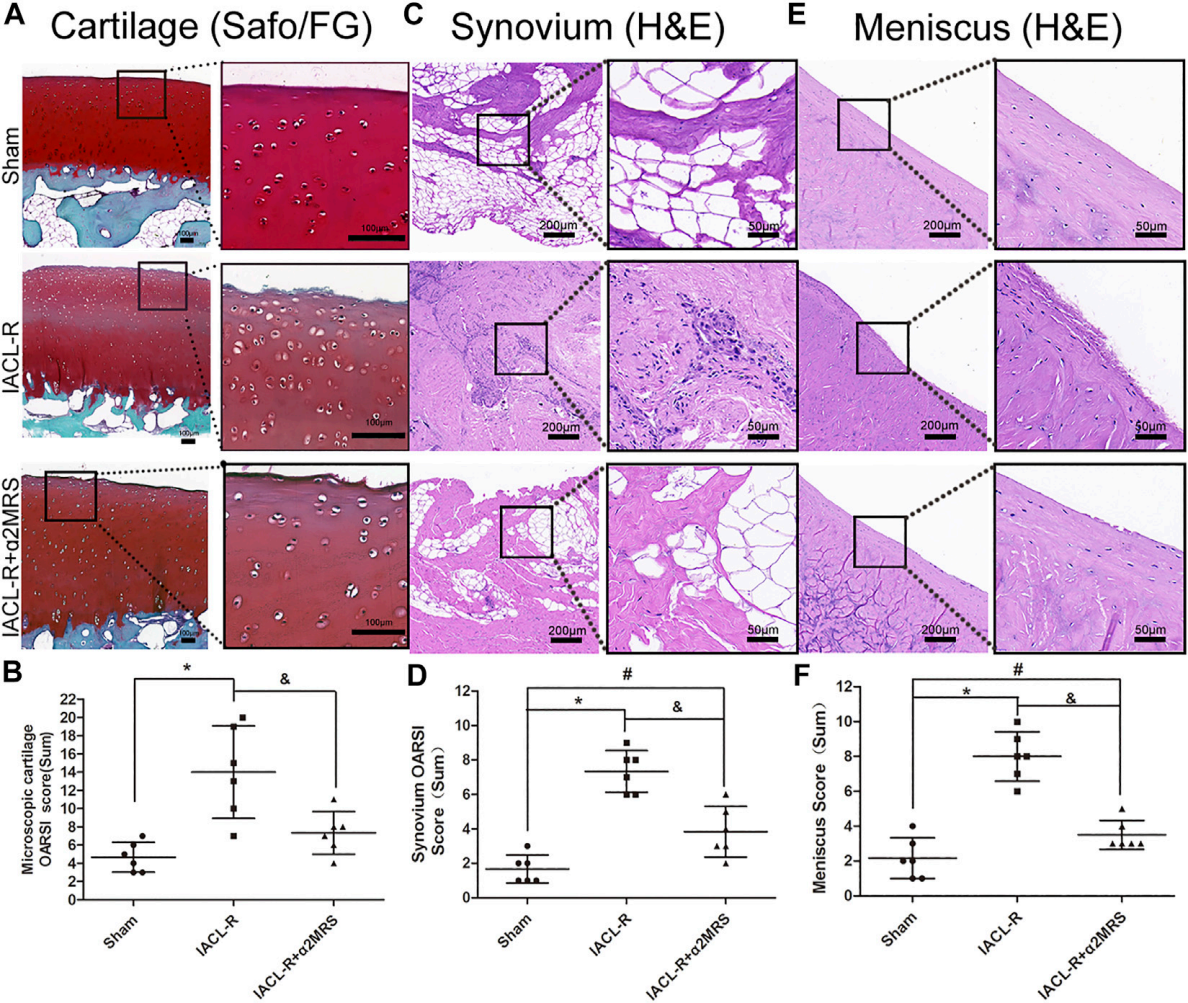
**(A)** Compared with the IACL-R group, less decrease in safranin O staining and surface fibrillation were observed in the IACL-R+α2MRS group. **(B)** Microscopic cartilage score were lower in the IACL-R+α2MRS group than in the IACL-R group (*p* = 0.015). **(C)** On H&E staining of the synovium, we found less degeneration in the IACL-R+α2MRS group than in the IACL-R group. **(D)** Microscopic synovium score were lower in the IACL-R+α2MRS group than in the IACL-R group (*p* = 0.002). **(E)** On H&E staining of the meniscus, we found less degeneration in the IACL-R+α2MRS group than in the IACL-R group **(F)** Microscopic meniscus score were lower in the IACL-R+α2MRS group than in the IACL-R group (*p* < 0.001). * = *p* < 0.05, sham group versus IACL-R group; # = *p* < 0.05, sham group versus IACL-R+α2MRS group; and = *p* < 0.05, IACL-R group versus IACL-R+α2MRS group. The bars show the mean ± SD (*n* = 6).

Less decreases in safranin O staining and surface fibrillation were observed in the IACL-R+α2MRS group as compared to the IACLR-group (Figure 8A). The microscopic OARSI cartilage scores were lower in the IACL-R+α2MRS group than in the IACL-R group (*p* = 0.015; Figure 8B; Supplementary Figures 2A–E; Supplementary Table S2). Based on H&E staining of the synovium, we found mild intimal thickening, low inflammatory cell infiltration, and slight sub-intimal fibrosis and vascularity in the IACL-R+α2MRS group, relative to those in the IACL-R group (Figure 8C). Thus, the microscopic OARSI synovium scores, both total scores (*p* = 0.002) and single indicator scores, showed that synovial damage was lower in the IACL-R+α2MRS group than in the IACL-R group (Figure 8D; Supplementary Figures S2F–I; Supplementary Table S2). H&E staining of the meniscus also revealed mild surface fibrillation, normal cell distribution, and a normal collagen fiber organization (Figure 8E), and the meniscus score was lower in the IACL-R+α2MRS group than in the IACL-R group (*p* < 0.001; Figure 8F; Supplementary Figures 2J–L; Supplementary Table S2).

### Immunohistochemical assessment

**FIGURE 9:**
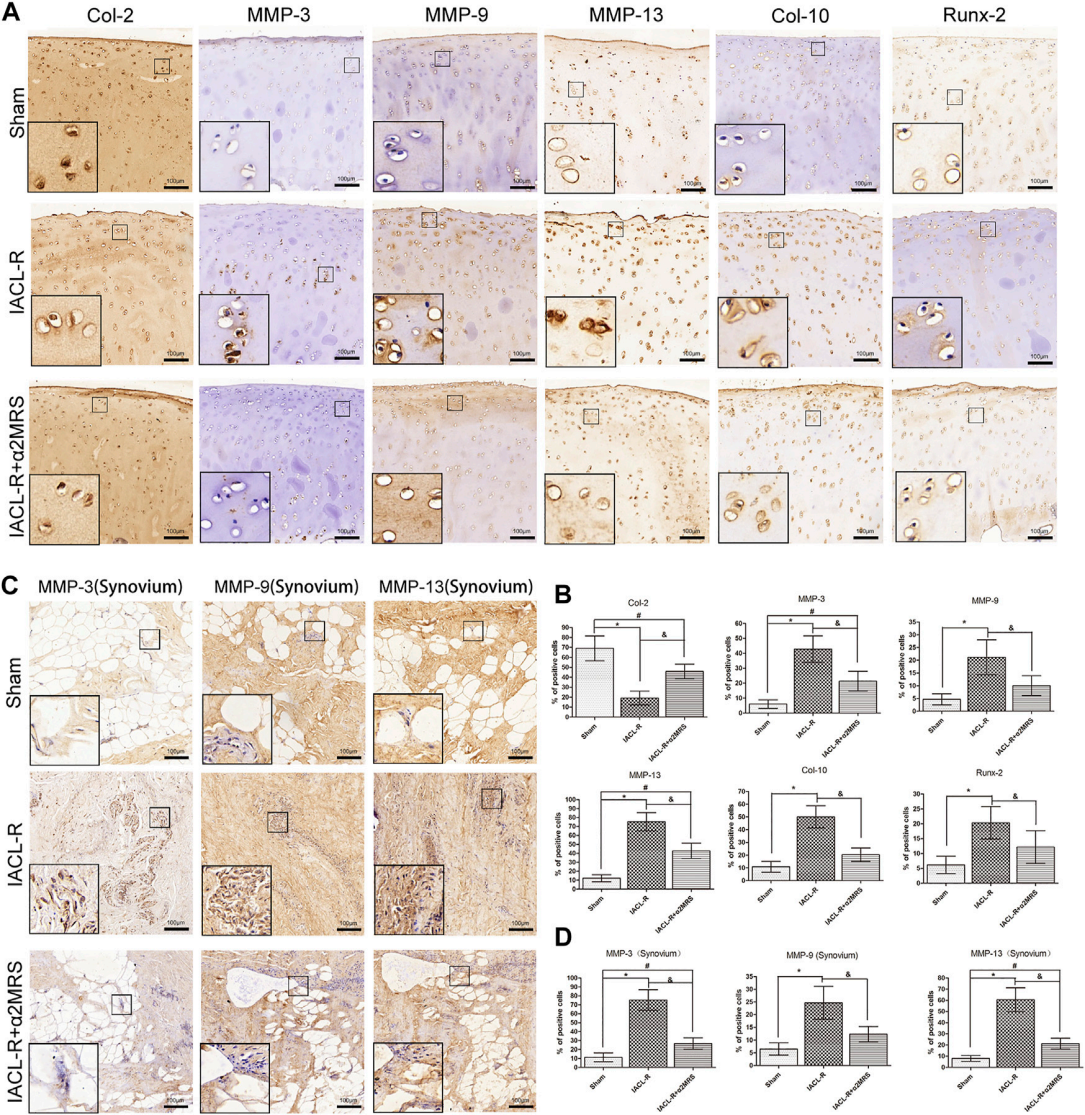
**(A)**: Articular cartilage immunostaining showed that MMP-3, MMP-9, MMP-13, Runx-2, and Col -10 staining significantly increased in the IACL-R mini pig group but were lower in the IACL-R+α2MRS group. In contrast, Col-2 expression in articular cartilage was higher in the IACL-R+α2MRS group than in the IACL-R group. **(B)** Quantitative immunohistochemical analysis of articular cartilage. **(C)** synovium immunostaining showed that MMP-3, MMP-9 and MMP-13 staining significantly increased in the IACL-R mini pig group but were lower in the IACL-R+α2MRS group. **(D)** Quantitative immunohistochemical analysis of synovium. * = *p* < 0.05, sham group versus IACL-R group; # = *p* < 0.05, sham group versus IACL-R+α2MRS group; and = *p* < 0.05, IACL-R group versus IACL-R+α2MRS group. The bars show the mean ± SD (*n* = 6).

Both articular cartilage (Figures 9A,B) and synovium (Figures 9C,D) immunostaining showed that MMP-3 (*p* < 0.001), MMP-9 (*p* < 0.001), MMP-13 (*p* < 0.001), Col -10 (*p* < 0.001), and Runx-2 (*p* = 0.001) staining significantly increased in the IACL-R group compared with the IACL-R+α2MRS group. In contrast, Col-2 (*p* < 0.001) expression in articular cartilage was higher in the IACL-R+α2MRS group than in the IACL-R group.

## Discussion

There are various conservative treatment methods for PTOA, including physical therapy, oral drugs, and intra-articular drug injection. Although physical therapy has the advantage of being non-invasive, it is mainly effective for patients with mild symptoms. Owing to the barrier function of the joint capsule, many oral drugs cannot enter the joint cavity to exert an effect. To date, intra-articular drug injection is the most effective method for the treatment of OA. α2M performs complex body functions, including the regulation of cytokine and hormone levels. It can bind several cytokines, including basic fibroblast growth factor, platelet-derived growth factor, nerve growth factor, IL-1β, and IL-6, and regulate the levels of hepcidin and leptin (Rehman et al., 2013). The specific mechanism of α2M has been previously reported by Sottrup-Jensen (Sottrup-Jensen 1989). Notably, recent studies showed that α2M can attenuate PTOA cartilage degeneration (Wang et al., 2014; Zhang et al., 2017; Li et al., 2019). However, α2M is not present in synovial fluid at sufficient levels due to its large molecular weight, which prevents its migration from the blood into the synovial fluid to counteract the increased concentrations of catabolic factors that appear after joint injury (Salvesen and Enghild, 1993). Thus, introducing supplemental α2M in the joint cavity might be a strategy to attenuate cartilage degeneration. However, considering the expense and potential safety concerns of biosynthetic α2M, α2MRS is a promising alternative for PTOA treatment. The results of our study demonstrate, for the first time, that α2MRS, as a master inhibitor of inflammatory factors, can attenuate cartilage degeneration *in vitro* and *in vivo*.

First, our *in vitro* data clearly demonstrated that human α2MRS promotes the proliferation of human chondrocyte, reduces the apoptosis of these, and decreases chondrocyte catabolic cytokine gene transcription and secretion, suggesting that α2MRS is a promising therapy. The ultimate goal of studying α2MRS is its clinical application. Thus, its biological safety and effectiveness need to be accurately evaluated and verified *in-vivo*.

Other studies have confirmed that significantly elevated inflammatory factors might be crucial for the pathogenesis of PTOA (Maerz et al., 2018; Zhao et al., 2021). There is a significant correlation between the expression of inflammatory factors, such as IL-1β, IL-6, and TNF-α, and cartilage injury (Han et al., 2018). Consistent with this, our findings demonstrated that different inflammatory factors exhibit different trends and could also play different pathogenic roles in the process of PTOA. Previous studies have demonstrated that the peak levels of cartilage catabolic enzymes could be detected on day 2 after joint injury (Zhao et al., 2021). Therefore, the mini pigs in the IACL-R+α2MRS group received the first α2MRS joint cavity injection 2 days after surgery, which inhibited the levels of various inflammatory factors. Luminex results revealed that not only the peak concentrations of inflammatory factors in synovial fluid were significantly reduced but that the speed of decline was also faster in the IACL-R+α2MRS group than in the IACL-R group, which demonstrated the early effect of α2MRS. In addition, gait analysis showed that the gait ratios of the left hind limb to the right hind limb of the IACL-R+α2MRS group were significantly greater than one at 7 days post-surgery, but it was significantly lower than that in the IACL-R group, which further proves the effectiveness of α2MRS. Other research results suggest that the activity of some cartilage catabolic enzymes might have two peaks. The first phase appears after the initial trauma to the joint. The second peak is associated with progressive cartilage degeneration at weeks four and 6 after surgery. Therefore, we repeated α2MRS supplementation, which constantly exerts an inflammation-inhibitory effect, and the inflammatory storm or waterfall effect was interrupted in time (Pan et al., 2016). Therefore, in the later stage (days 30-90) of the experiment, the concentration of inflammatory factors in the synovial fluid in the IACL-R+α2MRS group was maintained at a low level, and no obvious rebound was observed. Gait analysis is a relatively sensitive test for abnormal biomechanics and pain in the knee joint (Muramatsu et al., 2014; Capin et al., 2018; Hughes-Oliver et al., 2018). Compared with preoperative parameters, the gait of the IACL-R group also returned to initial levels, showing no significant difference in the middle stage (days 30-60). We conclude that the IACL-R model can restore the normal gait parameters of the knee joint and indirectly speculate that the ACL might have relatively stable biomechanical and consistent functions in different groups.

The mini pigs in the IACL-R+α2MRS group received α2MRS joint cavity injection four times starting from day 2 after surgery, which resulted in long-lasting inflammation suppression, significantly slowing the degeneration of articular cartilage. Therefore, the final imaging assessment, macroscopic cartilage assessment, and microscopic histological analysis confirmed that the articular cartilage in the IACL-R+α2MRS group was only slightly degenerated. Moreover, our biochemistry data demonstrated that supplemental α2MRS not only inhibited catabolic factors, including *MMP-3, MMP-13, Col-10,* and *Runx-2*, but also enhanced *Col-2* gene expression and protein synthesis. The increase in collagen suggests that α2MRS might have cartilage-repair functions. This finding is consistent with previous reports (Zhang et al., 2017). Moreover, the results of H&E staining of the synovium and meniscus proved that α2MRS injection into the joint cavity could significantly reduce inflammatory cell infiltration and vascularity, protecting the articular cartilage, synovium, and meniscus. Combining our results with those of other studies (Rehman, 2013), we speculate that α2MRS might act by binding cytokines, in addition to directly neutralizing enzyme activities, but the exact mechanism is not clear. The relative contributions of these mechanisms will be addressed in future studies.

Our study has a few limitations. First, the state of tension in the ACL or biomechanical changes in the knee joint post-surgery are crucial. To date, there is no technology or instrument that can accurately detect the smaller motions between the femur and tibia that are controlled by the ACL. The gait analysis used in this study can only roughly or indirectly assess the biomechanical stability of the knee joint. Second, although the concentration of α2M in α2MRS was 6.48-fold higher than that in normal mini pig serum, α2MRS is in fact a mixture containing extremely complex components. α2MRS was injected into the joint cavity, and proteins other than α2M might also play a role, but the exact mechanism is still unknown. Third, considering the side effects of the multiple anesthesia method used, we do not know the exact trend of inflammatory factor levels during the period from days 29–90 after surgery.

In summary, α2MRS is a promising bioinhibitor of catabolic proteases, and early and multiple injections in the joint cavity after IACL-R can significantly reduce the concentration of inflammatory factors in the joint synovial fluid of mini pigs and the degeneration of articular cartilage, exerting a chondroprotective effect.

## Data Availability

The original contributions presented in the study are included in the article/Supplementary Material further inquiries can be directed to the corresponding author.

## References

[B1] Barenius B. Sari P. Adel S. Robert B. Louise N. Karl E. (2014). Increased risk of osteoarthritis after anterior cruciate ligament reconstruction: A 14-year follow-up study of a randomized controlled trial. Am. J. Sports Med. 42 (5), 1049–1057. 10.1177/0363546514526139 24644301

[B2] Björnsson H. Samuelsson K. Sundemo D. Desai N. Sernert N. Rostgård-Christensen L. (2016). A randomized controlled trial with mean 16-year follow-up comparing hamstring and patellar tendon autografts in anterior cruciate ligament reconstruction. Am. J. Sports Med. 44 (9), 2304–2313. 10.1177/0363546516646378 27229354

[B3] Bryan J. Heard Y. A. Chung M. Nigel G. Shrive C. B. (2011). Early joint tissue changes are highly correlated with a set of inflammatory and degradative synovial biomarkers after ACL autograft and its sham surgery in an ovine model. J. Orthop. Res. 29 (8), 1185–1192. 10.1002/jor.21404 21387397

[B4] Capin J. J. Khandha A. Zarzycki R. Manal K. Buchanan T. S. Snyder-Mackler L. (2018). Gait mechanics after ACL reconstruction differ according to medial meniscal treatment. J. Bone Jt. Surg. Am. 100 (14), 1209–1216. 10.2106/JBJS.17.01014 PMC6636792 30020126

[B5] Chung T. J. Hsu K. Y. Chen J. H. Liu J. S. Chang H. W. Li P. F. (2016). Association of salivary alpha 2-macroglobulin levels and clinical characteristics in type 2 diabetes. J. Diabetes Investig. 7 (2), 190–196. 10.1111/jdi.12382 PMC4773666 27042270

[B6] Cuellar J. M. Cuellar V. G. Scuderi G. J. (2016). α2-Macroglobulin: Autologous protease inhibition technology. Phys. Med. Rehabil. Clin. N. Am. 27 (4), 909–918. 10.1016/j.pmr.2016.06.008 27788907

[B7] Gu X. D. Wei L. Li P. C. Che X. D. Zhao R. P. Han P. F. (2019). Adenovirus-mediated transduction with Histone Deacetylase 4 ameliorates disease progression in an osteoarthritis rat model. Int. Immunopharmacol. 75, 105752. 10.1016/j.intimp.2019.105752 31310910

[B8] Guo L. Wei X. Zhang Z. Wang X. Wang C. Li P. (2019). Ipriflavone attenuates the degeneration of cartilage by blocking the Indian hedgehog pathway. Arthritis Res. Ther. 21 (1), 109. 10.1186/s13075-019-1895-x 31046827 PMC6498579

[B9] Han P. F. Wei L. Duan Z. Q. Zhang Z. L. Chen T. Y. Lu J. G. (2018). Contribution of IL-1β, 6 and TNF-α to the form of post-traumatic osteoarthritis induced by "idealized" anterior cruciate ligament reconstruction in a porcine model. Int. Immunopharmacol. 65, 212–220. 10.1016/j.intimp.2018.10.007 30317108

[B10] Heard B. J. Solbak N. M. Chung M. Achari Y. Shrive N. G. Frank C. B. (2016). The infrapatellar fat pad is affected by injury induced inflammation in the rabbit knee: Use of dexamethasone to mitigate damage. Inflamm. Res. 65 (6), 459–470. 10.1007/s00011-016-0928-z 26898767

[B11] Ho A. S. Cheng C. C. Lee S. C. Liu M. L. Lee J. Y. Wang W. M. (2010). Novel biomarkers predict liver fibrosis in hepatitis C patients: Alpha 2 macroglobulin, vitamin D binding protein and apolipoprotein AI. J. Biomed. Sci. 17 (1), 58. 10.1186/1423-0127-17-58 20630109 PMC2914022

[B12] Hu W. Chen Y. Dou C. Dong S. (2020). Microenvironment in subchondral bone: Predominant regulator for the treatment of osteoarthritis. Ann. Rheum. Dis. 80 (4), 413–422. 10.1136/annrheumdis-2020-218089 33158879 PMC7958096

[B13] Hughes-Oliver C. N. Srinivasan D. Schmitt D. Queen R. M. (2018). Gender and limb differences in temporal gait parameters and gait variability in ankle osteoarthritis. Gait Posture 65, 228–233. 10.1016/j.gaitpost.2018.07.180 30558936

[B14] Kellgren J. H. Lawrence J. S. (1957). Radiological assessment of osteo-arthrosis. Ann. Rheum. Dis. 16 (4), 494–502. 10.1136/ard.16.4.494 13498604 PMC1006995

[B15] Li J. Kong X. B. Chen X. Y. Zhong W. Z. Chen J. Y. Liu Y. (2019). Protective role of α2-macroglobulin against jaw osteoradionecrosis in a preclinical rat model. J. Oral Pathol. Med. 48 (2), 166–173. 10.1111/jop.12809 30506608

[B16] Li L. Wei X. Wang D. Lv Z. Geng X. Li P. (2020). Positive effects of a young systemic environment and high growth differentiation factor 11 levels on chondrocyte proliferation and cartilage matrix synthesis in old mice. Arthritis Rheumatol. 72 (7), 1123–1133. 10.1002/art.41230 32067417

[B17] Li S. Xiang C. Wei X. Sun X. Li R. Li P. (2019). Early supplemental α2-macroglobulin attenuates cartilage and bone damage by inhibiting inflammation in collagen II-induced arthritis model. Int. J. Rheum. Dis. 22 (4), 654–665. 10.1111/1756-185X.13457 30609267 PMC6465088

[B18] Lieberthal J. Sambamurthy N. Scanzello C. R. (2015). Inflammation in joint injury and post-traumatic osteoarthritis. Osteoarthr. Cartil. 23 (11), 1825–1834. 10.1016/j.joca.2015.08.015 PMC4630675 26521728

[B19] Little C. B. Smith M. M. Cake M. A. Read R. A. Murphy M. J. Barry F. P. (2010). The OARSI histopathology initiative - recommendations for histological assessments of osteoarthritis in sheep and goats. Osteoarthr. Cartil. 18, S80–S92. 10.1016/j.joca.2010.04.016 20864026

[B20] Maerz T. Sherman E. Newton M. Yilmaz A. Kumar P. Graham S. F. (2018). Metabolomic serum profiling after ACL injury in rats: A pilot study implicating inflammation and immune dysregulation in post-traumatic osteoarthritis. J. Orthop. Res. 36 (7), 1969–1979. 10.1002/jor.23854 29315787

[B21] Misir A. Yildiz K. I. Kizkapan T. B. Incesoy M. A. (2020). Kellgren-Lawrence grade of osteoarthritis is associated with change in certain morphological parameters. Knee 27 (3), 633–641. 10.1016/j.knee.2020.04.013 32563417

[B22] Montesano G. Cuellar J. (2015). “Improving response to treatment for patients with DDD by the use of molecular markers,” in 2015. 4th International Conference on Orthopedics & Rheumatology, Baltimore, Maryland, USA, October 26-28, 2015.

[B23] Muramatsu Y. Sasho T. Saito M. Yamaguchi S. Akagi R. Mukoyama S. (2014). Preventive effects of hyaluronan from deterioration of gait parameters in surgically induced mice osteoarthritic knee model. Osteoarthr. Cartil. 22 (6), 831–835. 10.1016/j.joca.2014.03.016 24704496

[B24] Pan L. P. Cao Y. P. Wen L. C. Chai W. B. Jun-Bao D. U. Jin H. F. (2016). Hydrogen sulfide in cartilage and its inhibitory effect on matrix metalloproteinase 13 expression in chondrocytes induced by interlukin-1β. Beijing Da Xue Xue Bao Yi Xue Ban. 48 (2), 194–202. PMID: 27080266 27080266

[B25] Pauli C. Grogan S. P. Patil S. Otsuki S. Hasegawa A. Koziol J. (2011). Macroscopic and histopathologic analysis of human knee menisci in aging and osteoarthritis. Osteoarthr. Cartil. 19 (9), 1132–1141. 10.1016/j.joca.2011.05.008 PMC3217905 21683797

[B26] Pozzi F. Snyder-Mackler L. Zeni J. Jr. (2015). Relationship between biomechanical asymmetries during a step up and over task and stair climbing after total knee arthroplasty. Clin. Biomech. 30 (1), 78–85. 10.1016/j.clinbiomech.2014.11.001 PMC4298478 25467765

[B27] Rashid M. H. Theberge Y. Elmes S. J. Perkins M. N. Mcintosh F. (2013). Pharmacological validation of early and late phase of rat mono-iodoacetate model using the Tekscan system. Eur. J. Pain 17 (2), 210–222. 10.1002/j.1532-2149.2012.00176.x 22968802

[B28] Rehman A. A. Ahsan H. Khan F. H. (2013). α-2-Macroglobulin: A physiological guardian. J. Cell. Physiol. 228 (8), 1665–1675. 10.1002/jcp.24266 23086799

[B29] Ruan M. Z. C. Patel R. M. Dawson B. C. Jiang M. M. Lee B. H. L. (2013). Pain, motor and gait assessment of murine osteoarthritis in a cruciate ligament transection model. Osteoarthr. Cartil. 21 (9), 1355–1364. 10.1016/j.joca.2013.06.016 PMC3757305 23973150

[B30] Salvesen G. Enghild J. J. (1993). alpha-Macroglobulins: detection and characterization. Methods Enzymol. 223, 121–141. 10.1016/0076-6879(93)23041-k 7505874

[B31] Shah K. C. Linsley C. S. Wu B. M. (2020). Evaluation of a shape memory implant abutment system: An up to 6-month pilot clinical study. J. Prosthet. Dent. 123 (2), 257–263. 10.1016/j.prosdent.2018.11.012 31227233

[B32] Sottrup-Jensen L. (1989). Alpha-macroglobulins: Structure, shape, and mechanism of proteinase complex formation. J. Biol. Chem. 264 (20), 11539–11542. 2473064

[B33] Wang L. J. Zeng N. Yan Z. P. Li J. T. Ni G. X. (2020). Post-traumatic osteoarthritis following ACL injury. Arthritis Res. Ther. 24 (1), 57. 10.1186/s13075-020-02156-5 PMC7092615 32209130

[B34] Wang S. Wei X. Zhou J. Zhang J. Li K. Chen Q. (2014). Identification of α2-macroglobulin as a master inhibitor of cartilage-degrading factors that attenuates the progression of posttraumatic osteoarthritis. Arthritis Rheumatol. 66 (7), 1843–1853. 10.1002/art.38576 24578232 PMC4187342

[B35] Wei L. Fleming B. C. Sun X. Teeple E. Wu W. Jay G. D. (2010). Comparison of differential biomarkers of osteoarthritis with and without posttraumatic injury in the Hartley Guinea pig model. J. Orthop. Res. 28 (7), 900–906. 10.1002/jor.21093 20108346 PMC2875364

[B36] Xue Y. Chen Y. Jiang D. Wang L. Wang X. Li M. (2021). Self-reported weather sensitivity is associated with clinical symptoms and structural abnormalities in patients with knee osteoarthritis: A cross-sectional study. Rheumatol. Ther. 8 (3), 1405–1417. 10.1007/s40744-021-00340-w 34389921 PMC8380616

[B37] Zhang Y. Wei X. Browning S. Scuderi G. Hanna L. S. Wei L. (2017). Targeted designed variants of alpha-2-macroglobulin (A2M) attenuate cartilage degeneration in a rat model of osteoarthritis induced by anterior cruciate ligament transection. Arthritis Res. Ther. 19 (1), 175. 10.1186/s13075-017-1363-4 28743292 PMC5526282

[B38] Zhao R. Dong Z. Wei X. Gu X. Han P. Wu H. (2021). Inflammatory factors are crucial for the pathogenesis of post-traumatic osteoarthritis confirmed by a novel porcine model: "Idealized" anterior cruciate ligament reconstruction" and gait analysis. Int. Immunopharmacol. 99, 107905. 10.1016/j.intimp.2021.107905 34242997

