# Supplementary material for: α2-macroglobulin-rich serum as a master inhibitor of inflammatory factors attenuates cartilage degeneration in a mini pig model of osteoarthritis induced by “idealized” anterior cruciate ligament reconstruction
Source: Front Pharmacol. 2022 Sep 5;13:849102. doi: 10.3389/fphar.2022.849102 (PMC9483147; doi:10.3389/fphar.2022.849102)
Supplement: Supplementary file 1 [file DataSheet1.DOCX]

Supplementary Material

# Supplementary Text

**Supplementary Text 1:**

All minipigs underwent a minimum quarantine and stabilization period of 7 days. To minimize the animals’ distress, all operations strictly adhered to the principle of aseptic technique. All animals were fasted for 12 h before the surgery. The ability of the animals to obtain food and water was monitored daily. The research staff monitored the animals every day for the first 7 days and 3 days a week thereafter while the animals were located at the animal care facility. We evaluated the surgical knees for the presence of an infection. If signs of discomfort, swelling, or redness were found, a veterinarian was consulted to determine the appropriate antibiotic treatment. The animals were monitored to ensure that they were bearing weight. If an animal was not bearing weight after 2 weeks, the veterinarian was consulted to determine the appropriate course of action. In addition, the veterinarian was always consulted if pain was suspected; in this case, the animal would be further evaluated and treated to alleviate the pain or would be euthanized. The minimum pen size was 2.2 m^2^. All animals were allowed to drink freely and regular dosing.

**Supplementary Text 2:**

Before the MRI, the Kirschner wire was removed to avoid interference of the metal artifacts and ensure the integrity of the joint capsule. Furthermore, only the medial compartment of the right hind limb was subjected to MRI. The specific imaging parameters are listed below.

(1) X-ray examination: Faxitron UltraFocus, Tucson, AZ, USA; voltage, 37 KV; time, 4 s. Semi-flexed anterior-posterior and lateral views were taken in the unloaded state.

(2) CT examination: GE Revolution 256-row spiral CT; tube voltage, 120 KV; pitch, 0.992:1; thickness, 0.625 mm; 3D reconstruction workstation, GE AW4.7.

(3) MRI: DISCOVERY*MR 750w GE; field strength, 3.0 T

1. OSag fs PD: slice thickness, 2 mm; spacing, 0.2 mm; slices, 19; field of view, 20 × 20 cm; number of excitations, 4; acquisition time, 4.09 min;
2. Osag T2MAP: slice thickness, 3 mm; spacing, 1.5 mm; slices, 9; field of view, 20 × 20 cm; number of excitations, 2; acquisition time, 5.28 min.

# Supplementary Figures


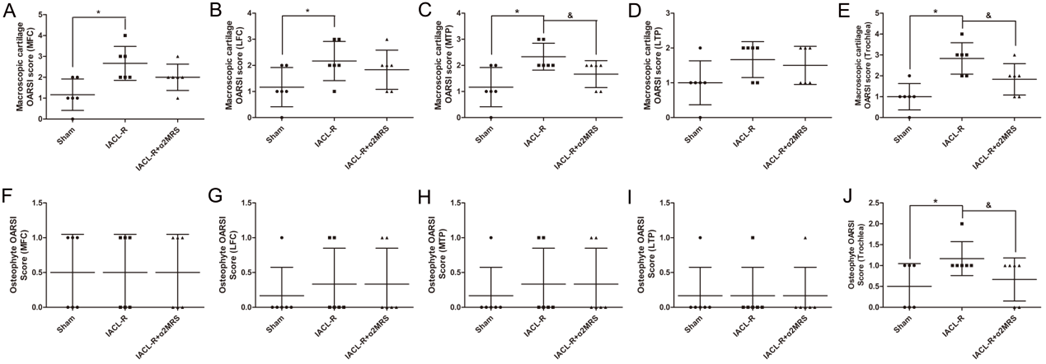


**Supplementary figure** **1(A-E)**: Macroscopic cartilage OARSI scores of the MTP and trochlea were significantly lower in the IACL-R+α2MRS group than in the IACL-R group (P<0.05). However, the macroscopic cartilage OARSI scores of the MFC, LFC and LTP did not differ between the IACL-R and IACL-R+α2MRS groups (P>0.05).Supplementary **figure 1(F-J)**: Osteophyte OARSI scores of the MFC, LFC, MTP, and LTP did not differ between the IACL-R and IACL-R+α2MRS groups (P>0.05). Only the score of the trochlea was significantly lower in the IACL-R+α2MRS group than in the IACL-R group (P<0.05). The bars show the mean ± SD. *=P < 0.05, sham group versus IACL-R group; &=P < 0.05, IACL-R group versus IACL-R+α2MRS group.MFC, medial femoral condyle; LFC, lateral femoral condyle; MTP, medial tibial plateau; LTP, lateral tibial plateau.


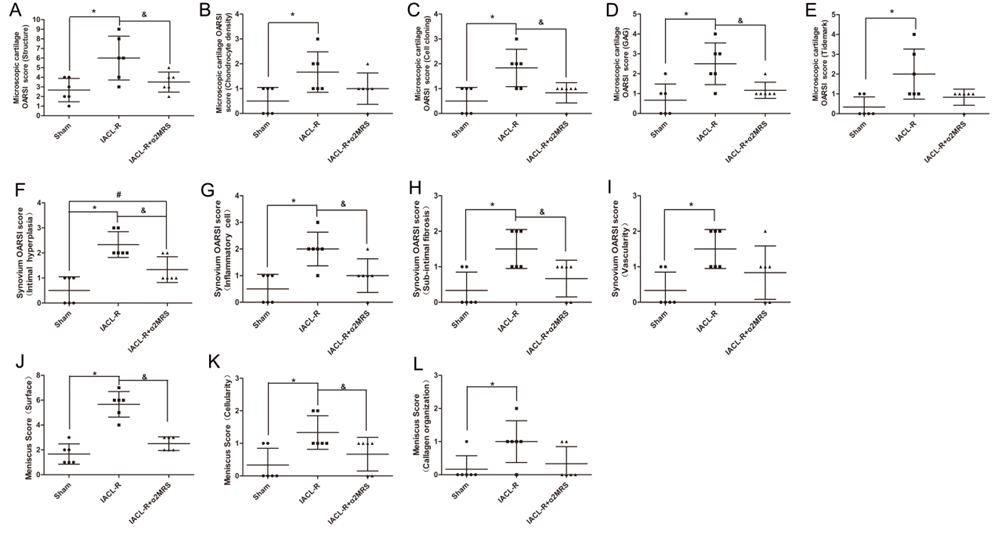
 **Supplementary figure 2(A-E)**: Microscopic cartilage OARSI scores of the structure, chondrocyte cloning and GAG were significantly lower in the IACL-R+α2MRS group than in the IACL-R group (P<0.05). However, the microscopic cartilage OARSI scores of the chondrocyte density and tidemark did not differ between the IACL-R and IACL-R+α2MRS groups (P>0.05).Supplementary **figure 2(F-I)**: Microscopic OARSI synovium scores of the intimal hyperplasia, inflammatory cell and sub-intimal fibrosis were significantly lower in the IACL-R+α2MRS group than in the IACL-R group (P<0.05). The microscopic synovium OARSI scores of the vascularity did not differ between the IACL-R and IACL-R+α2MRS groups (P>0.05).Supplementary **figure 2(J-L)**: The meniscus score of the surface fibrillation and cellularity were significantly lower in the IACL-R+α2MRS group than in the IACL-R group (P<0.05). The meniscus score of the collagen organization did not differ between the IACL-R and IACL-R+α2MRS groups (P>0.05). The bars show the mean ± SD. *=P < 0.05, sham group versus IACL-R group; #=P < 0.05, sham group versus IACL-R+α2MRS group; &=P < 0.05, IACL-R group versus IACL-R+α2MRS group.GAG, glycosaminoglycan.

# Supplementary Tables

| **Supplementary table 1**  **Primers used in this paper with their species, name, orientation, and sequence used in the RT-PCR protocol** | | | |
| --- | --- | --- | --- |
| Species | Name | Forward/reverse | Sequence |
| Human | Col-2 | Forward | CATGAGGGCGCGGTAGAGA |
|  | Col-2 | reverse | CCGGCTTCCACACATCCTTAT |
|  | Aggrecan | Forward | TGGTGATGATCTGGCACGAG |
|  | Aggrecan | reverse | CTCCGCTTCTGTAGTCTGCG |
|  | MMP-3 | Forward | CGCCAGACAAATGTGACCCT |
|  | MMP-3 | reverse | TACGGTTGGGAAGTTCTGGC |
|  | MMP-13 | Forward | CAGCCAACTGTGATCCTGCT |
|  | MMP-13 | reverse | CTTCATATGCGGCATCCACG |
| Minipig | Col-2 | Forward | \| ATTGCCTACCTGGACGAAGC \| \| --- \| |
|  | Col-2 | reverse | CGAGAGGTCTTCTGTGACCG |
|  | Aggrecan | Forward | \| \| ACCTAGTTTCGAGTGCCACG \| \| --- \| \| \| --- \| --- \| |
|  | Aggrecan | reverse | TCTTCTTTCCGGGTCGTTGG |
|  | MMP-3 | Forward | \| TGTTGCTGCCCATGAACTTG \| \| --- \| |
|  | MMP-3 | reverse | ACCGAGCCAGGTCTGTAAGT |
|  | MMP-13 | Forward | \| TCGGCTTAGAGGTGACTGGA \| \| --- \| |
|  | MMP-13 | reverse | CACATCAGGGACCCCACATC |
|  | Col-10a1 | Forward | \| AACGGGCAACAGCACTATGA \| \| --- \| |
|  | Col-10a1 | reverse | GAAGCCTGATCCAGGTAGCC |
|  | Runx-2 | Forward | \| ACAGAGTCAGATTACAGACCCCA \| \| --- \| |
|  | Runx-2 | reverse | GGTGGCAGTGTCATCATCTGAAAT |

**Supplementary table 2**

**WORMS , macroscopic cartilage score, osteophyte score ,microscopic cartilage score, synovium score and meniscus score**

| Parameter | Sham | IACL-R | IACL-R+  α2MRS | P value |
| --- | --- | --- | --- | --- |
|  | Median  (Q1, Q3) | Median  (Q1, Q3) | Median  (Q1, Q3) |  |
| WORMS sum (0–12) | 2  (1.5, 2.5) | 4.75  (4, 5.63) | 3  (2,4) | *& |
| MFC score (0–6) | 1  (0.75, 1.25) | 2.5  (2, 3) | 1.5  (1,2) | *& |
| MTP score (0–6) | 1  (0.75, 1.25) | 2.25  (2, 2.63) | 1.5  (1,2) | *& |
| Macroscopic cartilage  OARSI score (0–20) | 5  (3.25, 8.5) | 11.5  (9.5, 14.25) | 8.5  (7.75,9.5) | *#& |
| MFC score (0–4) | 1  (0.75, 2) | 2.5  (2, 3.25) | 2  (1.75,2.25) | * |
| LFC score (0–4) | 1  (0.75, 2) | 2  (1.75, 3) | 2  (1,2.25) | * |
| MTP score (0–4) | 1  (0.75, 2) | 2  (2, 3) | 2  (1,2) | *& |
| LTP score (0–4) | 1  (0.75, 1.25) | 2  (1, 2) | 1.5  (1,2) |  |
| Trochlea score (0–4) | 1  (0.75, 1.25) | 3  (2, 3.25) | 2  (1,2.25) | *& |
| Osteophyte OARSI  score (0–15) | 2  (0.75, 2) | 3  (1.75, 3) | 2.5  (0.75,3) |  |
| MFC score (0–4) | 0.5  (0, 1) | 0.5  (0, 1) | 0.5  (0, 1) |  |
| LFC score (0–4) | 0  (0,0.25) | 0  (0, 1) | 0  (0, 1) |  |
| MTP score (0–4) | 0  (0,0.25) | 0  (0, 1) | 0  (0, 1) |  |
| LTP score (0–4) | 0  (0,0.25) | 0  (0,0.25) | 0  (0,0.25) |  |
| Trochlea score (0–4) | 0.5  (0, 1) | 1  (1, 1.25) | 1  (0, 1) | *& |
| Microscopic cartilage  OARSI score (0–23) | 4.5  (3,6.25) | 14  (9.25,19.25) | 7.5  (5.75,8.75) | *& |
| Structure score (0–10) | 2.5  (1.75, 4) | 6  (3.75, 8.25) | 3.5  (2.75，4.25） | *& |
| Chondrocyte density score  (0–4) | 0.5 (0, 1) | 1.5  (1, 2.25) | 1  (0.75,1.25) | * |
| Cell cloning score (0–4) | 0.5  (0, 1) | 2  (1, 2.25) | 1  (0.75,1) | *& |
| GAG score (0–4) | 0.5  (0, 1.25) | 2.5  (1.75, 3.25) | 1  (1,1.25) | *& |
| Demark score (0–3) | 0  (0, 1) | 1.5  (1, 3.25) | 1  (0.75,1) | * |
| Synovium  OARSI score(0-12) | 1.5  (1,2.25) | 7.5  (6,8.25) | 3.5  (2.75,5.25) | *#& |
| Intimal hyperplasia score  (0-3) | 0.5  (0,1) | 2  (2,3) | 1  (1,2) | *#& |
| Inflammatory cell score  (0-3) | 0.5  (0,1) | 2  (1.75,2.25) | 1  (0.75,1.25) | *& |
| Sub-intimal fibrosis score  (0-3) | 0  (0,1) | 1.5  (1,2) | 1  (0,1) | *& |
| Vascularity score(0-3) | 0  (0,1) | 1.5  (1,2) | 1  (0,1.25) | * |
| Meniscus score(0-15) | 2  (1,3.25) | 8  (6.75,9.25) | 3  (3,4.25) | *#& |
| Surface score (0-9) | 1.5  (1,2.25) | 6  (4.75,6.25) | 2.5  (2,3) | *& |
| Cellularity score (0-3) | 0  (0,1) | 1  (1,2) | 1  (01) | *& |
| Collagen  organization score (0-3) | 0  (0.0.25) | 1  (0.75,1.25) | 0  (0,1) | * |

Data are presented as median (Q1, Q3), n = 6.

MFC, medial femoral condyle; LFC, lateral femoral condyle; MTP, medial tibial plateau; LTP, lateral tibial plateau; GAG, glycosaminoglycan. *=P < 0.05, sham group versus IACL-R group, #=P < 0.05, sham group versus IACL-R+α2MRS group, &=P < 0.05, IACL-R group versus IACL-R+α2MRS group
